# Litter Size Variation in Hypothalamic Gene Expression Determines Adult Metabolic Phenotype in Brandt's Voles (*Lasiopodomys brandtii*)

**DOI:** 10.1371/journal.pone.0019913

**Published:** 2011-05-26

**Authors:** Xue-Ying Zhang, Qiang Zhang, De-Hua Wang

**Affiliations:** 1 State Key Laboratory of Integrated Management of Pest Insects and Rodents, Institute of Zoology, Chinese Academy of Sciences, Beijing, China; 2 Graduate School of Chinese Academy of Sciences, Beijing, China; Universidad Europea de Madrid, Spain

## Abstract

**Background:**

Early postnatal environments may have long-term and potentially irreversible consequences on hypothalamic neurons involved in energy homeostasis. Litter size is an important life history trait and negatively correlated with milk intake in small mammals, and thus has been regarded as a naturally varying feature of the early developmental environment. Here we investigated the long-term effects of litter size on metabolic phenotype and hypothalamic neuropeptide mRNA expression involved in the regulation of energy homeostasis, using the offspring reared from large (10–12) and small (3–4) litter sizes, of Brandt's voles (*Lasiopodomys brandtii*), a rodent species from Inner Mongolia grassland in China.

**Methodology/Principal Findings:**

Hypothalamic leptin signaling and neuropeptides were measured by Real-Time PCR. We showed that offspring reared from small litters were heavier at weaning and also in adulthood than offspring from large litters, accompanied by increased food intake during development. There were no significant differences in serum leptin levels or leptin receptor (OB-Rb) mRNA in the hypothalamus at weaning or in adulthood, however, hypothalamic suppressor of cytokine signaling 3 (SOCS3) mRNA in adulthood increased in small litters compared to that in large litters. As a result, the agouti-related peptide (AgRP) mRNA increased in the offspring from small litters.

**Conclusions/Significance:**

These findings support our hypothesis that natural litter size has a permanent effect on offspring metabolic phenotype and hypothalamic neuropeptide expression, and suggest central leptin resistance and the resultant increase in AgRP expression may be a fundamental mechanism underlying hyperphagia and the increased risk of overweight in pups of small litters. Thus, we conclude that litter size may be an important and central determinant of metabolic fitness in adulthood.

## Introduction

Early-life environmental influences on the adult metabolic phenotype are of interest both scientifically and clinically, as it relates to the risk factors contributing to the obesity epidemic [1]. Epidemiological and experimental studies show a linkage between low birth weight and increased obesity [2], which implies the importance of intrauterine environment in remodeling adult phenotypes. In contrast, rapid growth during lactation also increases obesity risk. For instance, maternal high-fat diet during lactation can induce offspring insulin resistance and obesity in adulthood [3]. Adult rats [4], [5] and mice [6], [7] previously subjected to early postnatal overnutrition in small litters are hyperphagic, hyperleptinemic and differ in emotional behavior from control litters. These observations from either maternal high-fat diet or litter size manipulation underscore the critical importance of early postnatal nutritional environment in “programming” the long-term regulation of energy homeostasis [8], [9].

Litter size is an important life history trait [10], which is correlated negatively with postnatal growth [11], [12] and thus may determine an individual's reproductive success, longevity or other fitness-correlated traits [13]. Despite its importance in evolution, few studies have been made to investigate the physiology and central mechanisms contributing to the long-term effect of litter size on adult metabolic phenotype. We utilized seasonal breeding Brandt's voles (*Lasiopodomys brandtii*), which have a mean litter size of seven (litter size varies from 2 to 14) [14]. In a previous study, we found that the pup mass at birth was not related to litter size, however, the offspring raised in litter size of four were 18% heavier than those from a litter size of ten at peak lactation in the voles [15]. Therefore, the offspring raised in different natural litter sizes are an appropriate model to study consequences of nutritional variations during the critical postnatal period on the regulation of adult energy homeostasis.

The mediobasal hypothalamus is the site of energy homeostasis, and can exquisitely sense and integrate peripheral metabolic cues to coordinate peripheral metabolism [16]. Adipose-tissue derived leptin represents one important metabolic signal which acts on the hypothalamus. In response to feeding, leptin is secreted from adipose tissue and engages leptin receptors (OB-Rb) in various hypothalamic regions, leading to activation of the JAK2-STAT3 pathway and increased metabolic rate and decreased food intake via a reduction in orexigenic neuropeptide Y (NPY) and agouti-related peptide (AgRP), and increase expression of anorexigenic proopiomelanocortin (POMC) and cocaine- and amphetamine-regulated transcript (CART) [17], [18], [19]. Meanwhile, the JAK2–STAT3 pathway stimulates transcription of suppressor of cytokine signaling 3 (SOCS3), a negative regulator of leptin signaling following Ob-Rb activation [20]. The decreased activation of leptin signaling and the increase in hypothalamic SOCS3 expression have been clearly related with leptin resistance in obesity [21], [22].

In the present study, we investigated the long-term effects of natural litter sizes on offspring metabolic phenotype and biomarkers, such as food intake, resting metabolic rate (RMR), nonshivering thermogenesis (NST), uncoupling protein 1 (UCP1) in brown adipose tissue (BAT), body compositions, serum leptin and tri-iodothyronine (T3) and thyroxine (T4) levels in adulthood. In addition, we analyzed gene expression of OB-Rb, SOCS3, and orexigenic and anorexigenic neuropeptides in hypothalamus from young and adult voles raised in large and small litters. We hypothesized that postnatal litter size would permanently influence offspring metabolic phenotype and hypothalamic neuropeptide expression. We predicted that offspring reared from small litters would exhibit hyperphagia, excessive weight gain and hyperleptinemia in adulthood as compared to the counterparts from large litters, and that these phenotypes in small litters would be related to greater expression in hyperthalamic orexigenic neuropeptides.

## Materials and Methods

### Ethics Statement

All experimental protocols were reviewed and approved by the Animal Care and Use Committee of Institute of Zoology, the Chinese Academy of Sciences. The institute does not issue a number to any animal study, but each study requires the permit to use animals from the ethical committee. The animal facility must be licensed by the experimental animal committee of Beijing, and all staff, fellows and students must receive appropriate training before performing animal studies.

### Animals

Brandt's voles were the offspring of our laboratory breeding colony founded by field-captured animals. After weaning (21 days of age), voles were housed as same gender sibling pairs in plastic cages (30×5×20 cm) and maintained in temperature (23±1°C) and humidity-controlled rooms under a 16∶8 h light/dark photoperiod with lights on at 04:00 h. All animals were provided standard rabbit pellet chow (KeAo Feed Co., Beijing) and water *ad libitum*.

At 3–4 months of age, virgin female voles were housed individually and acclimated for 2 weeks and then were paired with males for 4 days to allow mating. On the day of parturition, the dams with 10–12 pups (regarded as large) and 3–4 pups (regarded as small) were selected to compare the effect of litter size, since we found previously that there was a difference in pup body mass only between these two groups [15]. Four out of twelve dams in large litters and five out of seventeen dams in small litters killed some of their pups (5 pups were dead in either group). At weaning, one pup from every litter was sacrificed to collect tissues (without regard to gender), and another two (one male and one female) were housed individually until 13 weeks. During lactation, all the pups from one nest were weighed every 3 days. After weaning, body mass and food intake were recorded weekly. Food intake was determined for three consecutive days and the remains were collected after the 3-day test. RMR and NST were measured at 12 weeks of age. We compared the effects of litter size and gender in adulthood; therefore four groups (LM, male from large litter, *n* = 12; SM, male from small litter, *n* = 9; LF, female from large litter, *n* = 13; SF, female from small litter, *n* = 10) were used in this study.

All the animals were sacrificed by CO_2_ overdose between 09:00 h and 11:00 h at 13 weeks of age. Trunk blood was collected and centrifuged at 4000 rpm for 30 min at 4°C and serum stored at −80°C until assayed. The whole brains were rapidly removed and placed on dry ice for slow freezing. A slice of brain tissue was cut between the optic chiasm and the mammillary bodies, and the hypothalamus was dissected by one horizontal cut immediately below the anterior commissure and sagittal cuts through the edge of the septum and perihypothalamic sulcus as previously described [23]. The hypothalamus was frozen in liquid nitrogen immediately and stored at −80°C until subsequent analysis. The interscapular brown adipose tissue (iBAT) was immediately and carefully dissected, weighed and stored at −80°C until assayed.

### Metabolic trials

RMR and NST were measured by using an open-circuit respirometer (FOXBOX, Sable Systems International Inc., Las Vegas, NV, USA). To avoid possible effects of circadian rhythm interfering with the group effects, two groups of animals were measured in an alternating manner between 08:00 h and 17:00 h. RMR was assessed from the rate of O_2_ consumption and CO_2_ production at 30°C (within their thermal neutral zone) (constant-temperature incubator; model LRH-250; Yiheng Co., Shanghai, CHN). A vole was placed in a chamber (200×130×85 mm, volume 1.4 L) for 2 h. The flow rate of incurrent and excurrent air (dried with anhydrous CaSO_4_; W. A. Hammond Drierite Co., USA) was approximately 300–400·ml·min^–1^ and 100 ·ml·min^–1^, respectively. The baseline of oxygen and carbon dioxide concentration were measured before and after each test. Oxygen consumption was recorded at intervals of 10 s. RMR was estimated from the stable lowest consecutive rate of oxygen consumption over 5 min.

The voles stayed in the chamber for another 1 h for NST measurement. Maximum NST was induced by a subcutaneous injection of norepinephrine (NE) at 25±1°C and mass-dependent dosage of NE (Shanghai Harvest Pharmaceutical Co. LTD) was calculated according to Heldmaier [24] and the recommended dosage in Brandt's voles [25]. NST was calculated from the stable highest consecutive rate of oxygen consumption over 5 min. The rate of oxygen consumption was calculated according to the equation.

V_O2_ = 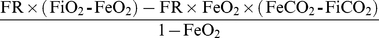 (FR = flow rate, V = exchange rate for the gas in question (O_2_, CO_2_), Fi = input fractional concentration, Fe = excurrent fractional concentration).

### Body composition analysis

After dissection of the hypothalamus and iBAT, the following organs and tissues, including the heart, lungs, liver, kidneys, spleen, gonad, stomach, small intestine, caecum, colon, together with subcutaneous fat, epididymal fat, mesenteric fat, epigonadal fat were extracted and weighed (±1 mg). The organs and carcass with fat pads were then dried in an oven at 60°C to constant weight. Body fat extraction from dry grinded carcass was performed with a Soxhlet Fat Extraction System (Avanti 2050; FOSS, Hogänäs, Sweden) with petroleum ether.

### Serum assays

Serum leptin levels were determined by radioimmunoassay (RIA) with the ^125^I Multi-species Kit (Cat. No. XL-85K, Linco Research Inc.), which had been validated in Brandt's voles [26], [27]. The lowest leptin level detected by this assay when using a 100 µl sample was 1.0 ng/ml. The intra- and inter-assay coefficients of variation were 3.6% and 8.7%, respectively.

Serum T3 and T4 were quantified using RIA kits (Institute of Chinese Atomic Energy, Beijing) according to the instructions and we have validated this kit for use in Brandt's voles previously [26]. Intra- and inter-assay coefficients of variation were 2.4% and 8.8% for the T3, and 4.3% and 7.6% for T4, respectively.

### Measurement of UCP1, COX4 and SIRT1 content in iBAT

Total protein content in iBAT was determined by Folin phenol method with bovine serum albumin as standard [28]. Uncoupling protein 1 (UCP1) and cytochrome c oxidase 4 (COX4), and SIRT1 content in iBAT was measured by Western blotting [27], [29]. Total iBAT protein (90 µg/lane) was separated in a discontinuous SDS-polyacylamide gel (12.5% running gel and 3% stacking gel for UCP1, COX4 and β-tubulin; 8% running gel and 3% stacking gel for SIRT1 and β-tubulin) and transferred onto PVDF membranes (Hybond-P; Amersham, Buckinghamshire, UK). After transfer, membranes were stained with Ponceau S to confirm equal loading and transfer. Membrane were then blocked in 5% milk in Tris-buffered saline-Tween for 1 h at room temperature and probed with the indicated antibodies overnight at 4°C. Following incubation with the appropriate horseradish peroxidase-conjugated secondary antibody for 1 h, the bands were visualized by chemiluminescence (Amersham Life Sciences, Little Chalfont, UK). Densitometry was performed using Quantity One (version 4.4.0) software (BioRad, Hercules, CA).

Primary antibodies used were as follows: rabbit anti-UCP1 (ab10983, Abcam, Cambridge, MA, USA), diluted 1∶10,000; mouse anti-COX4 (sc-58348, Santa Cruz Biotechnology, Inc., CA, USA), diluted 1∶1,000; rabbit anti-SIRT1 (H-300) (sc-15404, Santa Cruz Biotechnology, Inc.), diluted 1∶1,000; mouse anti-β-tubulin (E7, DSHB, Iowa City, Iowa, USA), diluted 1∶5000. The secondary antibodies of goat anti-rabbit IgG (1∶5,000; ZSGB-BIO Co., Beijing, CHN) and goat anti-mouse IgG (1∶5,000; ZSGB-BIO Co., Beijing, CHN) were used.

### Real-time PCR for measurement of hypothalamic OB-Rb, SOCS3, NPY, AgRP, POMC and CART mRNA expression

Total RNA was isolated using Trizol (Cat. No. 15596-026, Invitrogen, Carlsbad, CA, USA) following the manufacturer's instructions. After treating with DNase I (Cat. No. M6101, Promega, USA), RNA was reverse transcribed (4 µg total RNA) using a first-strand cDNA synthesis kit (Cat. No. 1622, Fermentas, Vilnius, the Republic of Lithuania).

Real time PCR reactions were performed in a 12.5 µL total volume comprised of 6.25 µL 2×SYBR Premix EX Taq™master mix, 1 µL cDNA templates and 0.2 µmoL/L primers using the SYBR Green I qPCR kit (Cat. No. DRR041D, TaKaRa, Shiga, Japan) in the Mx3005P quantitative PCR system (Stratagene, La Jolla, CA, USA). Thermal cycling conditions were: 95°C for 10 s, 40 cycles of 95°C for 5 s, 60°C for 20 s, and 72°C for 20 s. Samples were run in duplicate and all runs were accompanied by the housekeeping gene β-actin. Species-specific primers were designed (Table 1) and verified effectively in Brandt's voles [30]. Standard curves were constructed for each gene via serial dilutions of cDNA (1 to 26-fold dilutions). Analysis of standard curves between target genes and β-actin showed that they had similar amplification efficiency, which ensures the validity of the comparative quantity method. The data derived from Mx3005P quantitative software were expressed as relative amounts, which were calculated by normalizing the amount of target gene to β-actin mRNA levels. No amplification was detected in absence of template or in the no RT control.

**Table 1:** Gene-specific primers used for Real-Time PCR.

Primers	Forward primer (5′-3′)	Backward primer (5′-3′)	Product size (bp)
OB-Rb	CTGAGAGGGGTTCTCTTTGT	TCTTGCTCATCCTCCGTTTC	147
SOCS3	AGAAGATTCCGCTGGTACTG	GCTGGGTCACTTTCTCATAGG	114
NPY	TCGCTCTGTCCCTGCTCGTGTG	TCTCTTGCCGTATCTCTGCCTGGTG	116
AgRP	GCCCTGTTCCCAGAGTTCCC	ATCTAGGACCTCCGCCAAAGC	114
POMC	AAGATGGGCTCTACGGGATG	GTTCTTGACGATGGCGTTCT	134
CART	TGGAACCTGGCTTTAGCAAC	TACTCTGCACATGCCGACAC	145
β-actin	TTGTGCGTGACATCAAAGAG	ATGCCAGAAGATTCCATACC	200

Ob-Rb, leptin receptor; SOCS3, suppressor of cytokine signaling 3; NPY, neuropeptide Y; AgRP, agouti-related peptide; POMC, pro-opiomelanocortin; CART; cocaine- and amphetamine-regulated transcript.

### Statistical analysis

Data were analyzed using SPSS 13.0 (SPSS, Chicago, IL, USA). Prior to all statistical analyses, data were examined for assumptions of normality of variance using the Kolmogorov–Smirnov tests. Non-normally distributed data underwent logarithm or arcsine square root transformation. The temporal changes in body mass and food intake were assessed by repeated-measures ANOVA, followed by LSD post-hoc test. Group differences in body mass and food intake were assessed by two-way ANOVA and ANCOVA respectively. Differences among groups in serum leptin, T3, T4 levels, the mRNA levels of BAT UCP1, hypothalamic Ob-Rb, SOCS3, NPY, AgRP, POMC and CART in adult were assessed by two-way ANOVA, followed by Tukey post-hoc test. Differences in body compositions were analyzed by two-way ANCOVA with body mass as a covariate. At weaning, the differences in UCP1 and hypothalamic gene expression were assessed by independent samples t-test. Pearson correlation analyses were used to detect possible associations of serum leptin levels with body fat mass and food intake. Data are expressed as mean±SE. Values of P<0.05 were considered statistically significant.

## Results

### Body mass

**Figure 1:**
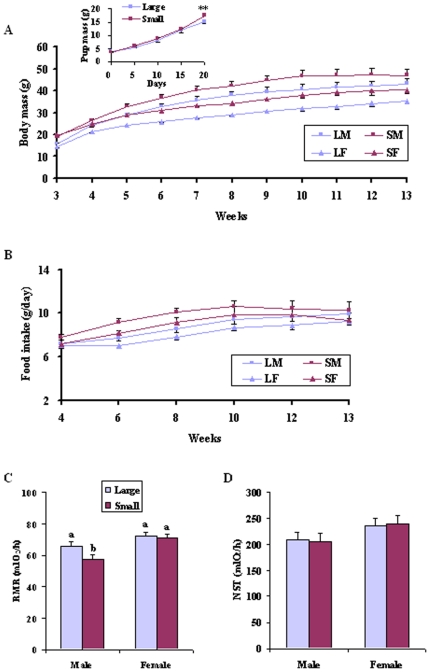
Body mass (A), food intake (B), RMR (C) and NST (D) throughout the experiment. The insert in panel 1A shows the pup mass during lactation (** P<0.01). Values are presented as mean±SE. Group differences are expressed as P<0.05, and bars with different letters differed significantly from each other. RMR, resting metabolic rate; NST, nonshivering thermogenesis.

At birth, there was no difference in body mass per pup between large and small litters (P>0.05; Fig. 1A insert). At weaning, the offspring from small litters were 29% heavier than pups from large litters (F_1, 42_ = 79.420, P<0.001) (Fig. 1A). At weeks 13, the offspring from small litters were 11% heavier than offspring from large litters (F_1, 42_ = 4.103, P<0.05), and the males were 20% heavier than females (F_1, 42_ = 12.023, P<0.001). After adjusting for the effect of body mass at weaning, body mass was no longer affected by litter size (P>0.05), but remained significantly influenced by gender (P<0.01) until the end of the experiment. During the course of experiment, all voles showed a continuous growth until weeks 10 or 11, after which, body mass stabilized (Repeated measure, LM, F_10, 110_ = 59.482, P<0.001; SM, F_10, 100_ = 62.200, P<0.001; LF, F_10, 120_ = 120.061, P<0.001; SF, F_10, 90_ = 69.369, P<0.001) (Fig. 1A).

### Food intake

Food intake was analyzed by ANCOVA with body mass as a covariate. At the age of 4 weeks, food intake was not affected by either litter size (F_1, 41_ = 2.682, P>0.05), gender (F_1, 41_ = 1.115, P>0.05), or their interaction (F_1, 41_ = 2.648, P>0.05) (Fig. 1B). During weeks 6 and 8, voles from small litters ate more food than those from large litters (P>0.05). However, there were no effects of litter size or gender on food intake between weeks 10-13 (P>0.05).

### RMR and NST

The females had markedly higher RMR (F_1, 41_ = 12.503, P<0.001; Fig. 1C) and 15% higher NST (F_1, 41_ = 3.602, P = 0.065; Fig. 1D) than males. No differences were found in RMR (F_1, 41_ = 4.605, P>0.05) or NST (F_1, 41_ = 0.004, P>0.05) between litter sizes. Males from small litters had lower RMR compared to any other group (F_3, 41_ = 5.433, P<0.01), but there was no significant difference in NST among groups (F_3, 41_ = 1.253, P>0.05).

### Serum leptin, T3 and T4 levels

**Figure 2:**
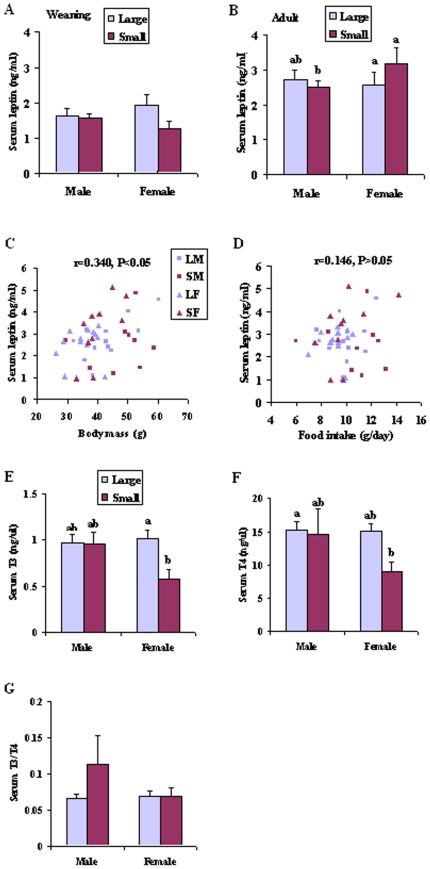
Serum leptin (A, weaning; B, adult), T3 (tri-iodothyronine, E) and T4 (thyroxine, F) levels in male and female voles from large and small litters. The adult serum leptin was correlated positively with body mass (C), but not with food intake (D). Values are presented as mean±SE. Group differences are expressed as P<0.05, and bars with different letters differed significantly from each other.

At weaning, there was no difference in serum leptin between litters (F_1, 39_ = 2.448, P>0.05) or gender (F_1, 39_ = 0.001, P>0.05) (Fig. 2A). Adult female voles had higher serum leptin levels compared to males (ANCOVA, F_1, 39_ = 5.340, P<0.05; Fig. 2B), but serum leptin in adulthood was not influenced by litter size (ANCOVA, F_1, 39_ = 0.171, P>0.05) (Fig. 2B). In adulthood, serum leptin was correlated positively with body mass (r = 0.340, P<0.05; Fig. 2C), but not with food intake (r = 0.146, P>0.05; Fig. 2D).

We did not measure serum T3 and T4 in the offspring at weaning because of small volume of serum. The adult offspring from small litters had lower T3 (F_1, 35_ = 4.367, P<0.05; Fig. 2E) and T4 levels (F_1, 35_ = 7.010, P<0.05; Fig. 2F) than from large, but the ratio of T3/T4 didn't differ between different litters (F_1, 35_ = 1.745, P>0.05; Fig. 2G). There was no difference between males and females for either T3 (F_1, 35_ = 2.466, P>0.05), T4 (F_1, 35_ = 1.042, P>0.05) or the ratio of T3/T4 (F_1, 35_ = 1.274, P>0.05).

### Biomarkers for thermogenesis in iBAT

**Figure 3:**
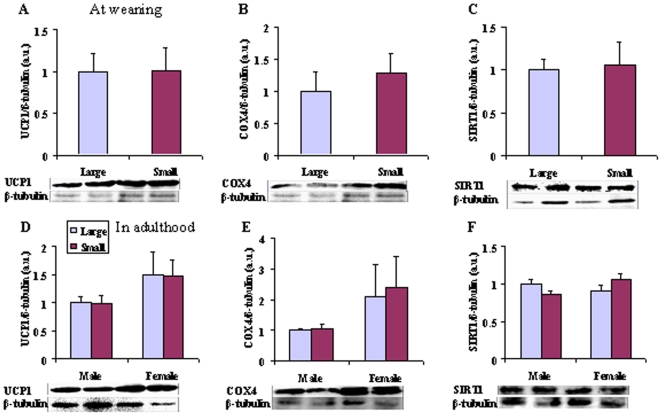
UCP1, COX4 and SIRT1 content in BAT at weaning (A–C) and in adulthood (D–F) in male and female voles from large and small litters. All these biomarkers in BAT were not affected by litter size, and the females showed more mRNA expression of UCP1 and COX4 in BAT than males. Values are presented as mean±SE. UCP1, uncoupling protein 1; COX4, cytochrome c oxidase 4; a.u., arbitrary unit.

At weaning (Fig. 3A–C), there was no difference in UCP1 (t = −0.060, df = 14, P>0.05; Fig. 3A), COX4 (t = −0.824, df = 14, P>0.05; Fig. 3B), and SIRT1 (t = −0.286, df = 14, P>0.05; Fig. 3C) protein content in iBAT between large and small litters. In adulthood (Fig. 3D–F), UCP1 and COX4 content remained unaffected by litter size (P>0.05), but females had 50% higher UCP1 (F_1, 28_ = 3.722, P = 0.064), and 26% higher COX4 content (F_1, 28_ = 2.924, P = 0.098) than males, which was in accordance with higher RMR and NST in females. Further, SIRT1 content in iBAT was influenced by the interaction of litter size and gender (F_1, 28_ = 4.572, P<0.05).

### Hypothalamic neuropeptide mRNA expression

There was no difference in hypothalamic OB-Rb, SOCS3, NPY, AgRP, POMC and CART mRNA expression between large and small litters at weaning (Table 2).

**Table 2:** Hypothalamic leptin signaling and neuropeptide expression between large and small litters at weaning.

	Large litter size (*n* = 10)	Small litter size (*n* = 9)	P
OB-Rb/β-actin	1.000±0.093	1.191±0.194	ns
SOCS3/β-actin	1.000±0.144	1.308±0.109	ns
NPY/β-actin	1.000±0.124	1.181±0.234	ns
AgRP/β-actin	1.000±0.166	1.162±0.151	ns
POMC/β-actin	1.000±0.163	1.146±0.151	ns
CART/β-actin	1.000±0.196	1.389±0.237	ns

Data are presented as means±SE. Values were analyzed by independent samples t-test, and P<0.05 was considered statistically significant. Ob-Rb, leptin receptor; SOCS3, suppressor of cytokine signaling 3; NPY, neuropeptide Y; AgRP, agouti-related peptide; POMC, pro-opiomelanocortin; CART; cocaine- and amphetamine-regulated transcript.

**Figure 4:**
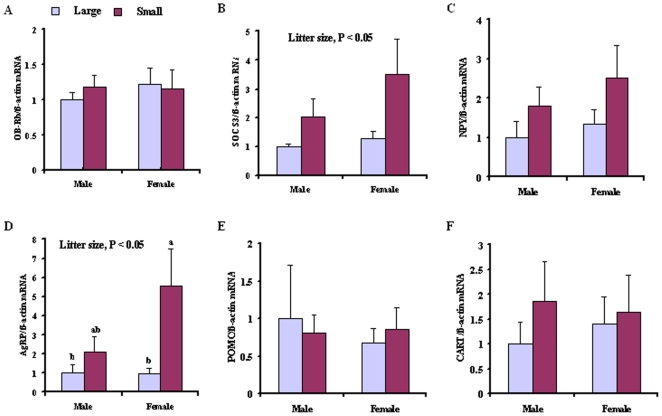
Hypothalamic OB-Rb (A), SOCS3 (B), NPY (C), AgRP (D), POMC (E) and CART (F) mRNA expression in adult male and female voles from large and small litters. Values are presented as mean±SE. Group differences are expressed as P<0.05, and bars with different letters differed significantly from each other. Ob-Rb, leptin receptor; SOCS3, suppressor of cytokine signaling 3; NPY, neuropeptide Y; AgRP, agouti-related peptide; POMC, pro-opiomelanocortin; CART; cocaine- and amphetamine-regulated transcript.

Likewise, in adults, OB-Rb mRNA expression in the hypothalamus was not affected by either litter size (F_1, 22_ = 3.600, P>0.05) or gender (F_1, 22_ = 0.560, P>0.05) (Fig. 4A). In contrast, the hypothalamic SOCS3 mRNA expression was greater in offspring from small litters, as compared to those from large litters (F_1, 22_ = 5.800, P<0.05) (Fig. 4B).

The orexigenic NPY mRNA level in small litters was 86% higher than in large litters, although the difference was not statistically significant (F_1, 22_ = 2.572, P>0.05 (Fig. 4C). Offspring from small litters had greater AgRP mRNA expression than did those from large litters (F_1, 22_ = 4.696, P<0.05), but there was no gender difference (F_1, 22_ = 1.700, P>0.05) (Fig. 4D) nor was there difference in anorexigenic neuropeptide (POMC and CART) expression by litter size or gender (P>0.05) (Fig. 4E, F).

### Body compositions

The data for organ mass are presented in Table 3. The offspring from small litters had a larger brain than those from large litters (F_1, 39_ = 14.592, P<0.001). In addition, BAT mass (F_1, 39_ = 12.016, P = 0.001) and dry stomach mass (F_1, 39_ = 5.131, P<0.05) were higher in females as compared to males. However, there were no differences observed in other organs between either litter sizes or gender.

**Table 3:** Dry mass of organs but brain and BAT (wet mass) in adult male and female offspring from large and small litters.

Parameters	Male		Female		Statistical summary		
(g)	Large (*n* = 12)	Small (*n* = 9)	Large (*n* = 13)	Small (*n* = 10)	Gender	Size	Gender×Size
Brain (wet)	0.540±0.015^b^	0.588±0.010^ab^	0.527±0.01^ab^	0.599±0.013^a^	ns	<0.001	ns
BAT (wet)	0.213±0.028	0.234±0.017	0.233±0.018	0.257±0.013	0.001	ns	ns
Heart	0.046±0.003	0.050±0.007	0.041±0.002	0.045±0.003	ns	ns	ns
Lungs	0.077±0.013	0.072±0.005	0.077±0.013	0.070±0.006	ns	ns	ns
Liver	0.541±0.119	0.564±0.062	0.477±0.033	0.459±0.036	ns	ns	ns
Kidney	0.115±0.007	0.117±0.009	0.089±0.003	0.102±0.005	ns	ns	ns
Spleen	0.008±0.001	0.008±0.001	0.007±0.001	0.008±0.001	ns	ns	ns
Uterus			0.035±0.005	0.030±0.004	-	ns	-
Testis	0.128±0.014	0.121±0.016			-	ns	-
Epididymis	0.023±0.004	0.023±0.003			-	ns	-
Seminal vesicle	0.121±0.028	0.102±0.019			-	ns	-
Stomach	0.057±0.002	0.065±0.004	0.056±0.002	0.066±0.004	<0.05	0.078	ns
Small intestine	0.104±0.012	0.079±0.007	0.069±0.004	0.073±0.009	ns	ns	ns
Cecum	0.078±0.008	0.067±0.004	0.068±0.007	0.067±0.007	ns	ns	ns
Colon	0.061±0.003	0.087±0.025	0.059±0.002	0.067±0.005	ns	ns	ns
Total gut mass	0.301±0.020	0.299±0.033	0.253±0.010	0.272±0.024	ns	ns	ns

Data are presented as means±SE. Values for a specific parameter that share different superscripts are significantly different at P<0.05, determined by a two-way ANCOVA with body mass as a covariate.

As compared to males, fhe females had more retroperitoneal (F_1, 39_ = 8.762, P<0.01), mesenteric (F_1, 39_ = 14.071, P = 0.001), perinephric (F_1, 39_ = 9.382, P<0.01) and total fat mass (F_1, 39_ = 10.347, P<0.01), but less epigonadal fat (F_1, 39_ = 6.186, P<0.01; Table 4). Offspring from small litters had slightly greater retroperitoneal fat mass than those from large litters (F_1, 39_ = 3.459, P = 0.070), and other fat pads and total fat mass did not vary with litter size (Table 4). Interestingly, offspring from small litters had higher carcass mass compared with those from large litters (wet mass, F_1, 39_ = 4.242, P<0.05; dry mass, F_1, 39_ = 3.703, P = 0.06), while females has less dry carcass mass than males (F_1, 39_ = 6.116, P<0.05; Table 4).

**Table 4:** Fat pads and carcass mass in adult male and female offspring from large and small litters.

Parameters	Male		Female		Statistical summary		
	Large (*n* = 12)	Small (*n* = 9)	Large (*n* = 13)	Small (*n* = 10)	Gender	Size	Gender×Size
Retroperitoneal fat (g)	0.259±0.061	0.239±0.096	0.275±0.051	0.284±0.062	<0.01	0.070	ns
Mesenteric fat (g)	0.207±0.016	0.203±0.015	0.199±0.010	0.233±0.020	0.001	ns	ns
Perinephric fat (g)	0.101±0.014	0.110±0.018	0.121±0.017	0.123±0.007	<0.01	ns	ns
Epigonadal fat (g)	0.523±0.063^ab^	0.483±0.089^a^	0.196±0.029^c^	0.271±0.062^bc^	<0.05	ns	ns
Total fat mass (g)	5.400±0.737^ab^	4.430±0.968^b^	5.026±0.522^a^	5.964±0.709^a^	<0.01	0.092	ns
Wet carcass mass (g)	30.271±1.827^a^	32.717±2.078^a^	24.280±1.172^b^	27.252±1.308^ab^	ns	<0.05	ns
Dry carcass mass (g)	12.728±1.021^ab^	12.595±1.227^a^	10.823±0.694^b^	12.026±0.471^ab^	<0.05	0.06	ns

Data are presented as means±SE. Values for a specific parameter that share different superscripts are significantly different at P<0.05, determined by a two-way ANCOVA with body mass as a covariate.

## Discussion

In this study, we used a wild rodent model to examine the consequences of postnatal litter size on offspring growth and adult metabolic phenotype, and to investigate the central mechanisms contributing to the long-term effect of litter size on metabolic fitness. As observed in several other rodent species [12], [31], [32], voles from small litters showed more rapid growth per pup during postnatal development than those from large litters. We also found that increases in hypothalamic SOCS3 and AgRP expression were associated with higher food intake and body mass in voles raised in small compared to large litters. These findings demonstrate that litter size may program adult metabolic phenotype by permanently influencing central leptin sensitivity and hypothalamic neuropeptide expression.

### Metabolic phenotype associated with different litter sizes

Consistent with studies in rats with natural [33] or manipulated litter sizes [34], [35] or maternal overnutrition [36], [37], Brandt's vole offspring from small litters were heavier at weaning, and remained heavier than those from large litters until the end of the experiment at 13 weeks of age, but there were no differences in peripheral and visceral fat pads between litter sizes. After adjusting for the effect of body mass at weaning, there was no difference observed in post-weaning body mass between different litter sizes. This suggests that the difference in adult body mass is totally determined by pre-weaning litter size. In addition, some studies suggest that maternal obesity interacts with post-weaning high-fat-diet consumption to cause greater adiposity [37]. However, the consequences resulting from the nutritional environment during lactation differ from the intrauterine environment. For example, intrauterine growth restriction may result in offspring catch-up growth during development and program obesity in adult [10], [38]. However, if malnutrition was prolonged throughout lactation, adult body weight can be permanently reduced [10]. These findings support the “thrifty phenotype hypothesis” generated by Hales and Barker [39], [40].

Although we did not measure locomotion, we found that higher energy intake contributed to the heavier body mass in the vole offspring from small litters. In a wide range of mammals, litter size is correlated negatively with milk intake and growth per pup during lactation [11], [15]. Even during post-weaning development, offspring from small litters still ate more compared to those from large litters, which is similar to the study in litter size-manipulated rats [34]. In the present study, we did not find any differences in protein levels of UCP1 (a molecular marker of BAT thermogenesis), COX4 (reflecting mitochondrial oxidative capacities) [41] and SIRT1 (evolutionarily conserved NAD+ dependent deacetylase regulating transcriptional networks in various critical metabolic processes) [42] in BAT, and in RMR and NST of the whole animal level between litter sizes. Moreover, thyroid hormone, especially the ratio of serum T3/T4, which is an important determinant of energy expenditure [43], [44], was not affected by litter size. Therefore, these results suggest that natural litter size did not induce long-term changes in energy expenditure in the voles. In contrast, when rats are raised in reduced nursing litter size, they demonstrated a reduction in cold-induced adaptive thermogenesis compared to controls [45]. The diverse results may attribute to the different models of early postnatal nutritional environment. Dams with manipulated litter sizes would have different energy output from those with the same natural litter size, thus the extent of malnutrition or overnutrition of the offspring in these models are different.

The similar energy intake, but higher RMR and NST associated with higher BAT mass, more UCP1 and COX4 content in BAT, may contribute to the lower body mass in the females as compared with the male voles. However, there was no gender difference in metabolic phenotype affected by litter size. This is in agreement with rat studies with different litter sizes or maternal nutrition [33], [36]. Thus, the effect of litter size on an animal's phenotype in adulthood is independent of gender.

### Central leptin sensitivity and hypothalamic neuropeptide expression programmed by litter size

In the present study, there was no difference in serum leptin levels at weaning or in adulthood between different litter sizes. This was also found in litter size-manipulated rats [46], whereas the rat offspring from undernutritioned dams throughout pregnancy showed hyperleptinemia in adulthood [47]. Similarly, this same phenomenon is observed in human studies with hyperleptinemia in infants from gestational undernutritioned or diabetic mothers [48]. Further, intracerebroventricular leptin administration to neonatal rats altered adult female phenotypes, including a reduction in body mass and food intake [49]. These studies suggest that perinatal leptin plays the critical role in programming adult metabolic phenotypes although serum leptin showed different responses to early nutritional environments.

Despite the similar levels in serum leptin and hypothalamic OB-Rb mRNA, we found that hypothalamic SOCS3 expression increased in the adult offspring from small litters, indicating central leptin resistance. Some recent studies in postnatal overfed rats and mice reported higher SOCS3 expression and lower STAT3 activity in adulthood [46], [50], similar to our present result in voles. Additionally, our findings are supported by another study which showed that the offspring from enlarged litter sizes had enhanced leptin sensitivity and were protected from obesity [35]. Indeed, leptin resistance was functionally verified by the absence of a decrease in food intake and body mass in response to leptin injection as well as by a lower expression of the hypothalamic leptin receptor and an increased expression of SOCS3 in neonatal leptin-treated [51], [52], [53] and maternal leptin-treated rats [54]. Interestingly, the rat offspring of intrauterine growth restriction also demonstrated leptin resistance, indicated by suppressed leptin-induced STAT phosphorylation [2]. These findings using diverse animal models imply the universality of both prenatal undernutrition and postnatal overnutrition resulting in impaired leptin signaling pathway and may explain the persistent hyperphagia and overweight observed in these models.

We further analyzed mRNA expression of hypothalamic neuropeptides related to orexigenic and anorexigenic pathways at weaning and in adulthood. We found increased mRNA expression of orexigenic AgRP and NPY (especially AgRP), but no changes in anorexigenic POMC and CART in the vole offspring from small litters in adulthood. Hypothalamic orexigenic NPY and AgRP mRNA expression also increased in high-fat fed rats from reduced litter size [55]. Moreover, the findings were partly supported by studies in maternal high-fat offspring which showed increased NPY immunoreactivity in arcuate nucleus at day 1 [56] or NPY Y1 receptor mRNA in the periventricular nucleus of the hypothalamus in adulthood [37]. Other orexigenic peptides, such as galanin, enkephalin and dynorphin in the paraventricular nucleus and orexin and melanin-concentrating hormone in the lateral hypothalamus, were found to increase in the offspring of rat dams on a high-fat diet during pregnancy [57]. These rats also exhibited an increase in neurogenesis in the hypothalamus, ultimately with a great proportion of new neurons expressed orexigenic peptides. All these findings indicate that hypothalamic neuropeptide expression may be programmed by nutritional environment induced by litter size during a critical window of postnatal development.

Taken together, voles from small litters showed greater food intake and body mass than voles from large litters. Litter size did not affect adult serum leptin levels, but did have long-term effects on hypothalamic leptin sensitivity. In addition, increased AgRP expression was associated with hyperphagia in the offspring from small litters. These findings provide further evidence that litter size can permanently influence leptin sensitivity and hypothalamic neuropeptides, resulting in bonafide changes in the adult metabolic phenotype. From a physiological point of view, this study also highlights the importance of litter size in evolution, and suggests that animals raised in natural litter sizes would not be susceptible to obesity unless subjected to artificial manipulation during the early postnatal period.

## References

[1] Cottrell EC, Ozanne SE (2007). Developmental programming of energy balance and the metabolic syndrome.. Proc Nutr Soc.

[2] Desai M, Gayle D, Han G, Ross MG (2007). Programmed hyperphagia due to reduced anorexigenic mechanisms in intrauterine growth-restricted offspring.. Reprod Sci.

[3] White CL, Purpera MN, Morrison CD (2009). Maternal obesity is necessary for programming effect of high-fat diet on offspring.. Am J Physiol Regul Integr Comp Physiol.

[4] Dimitsantos E, Escorihuela RM, Fuentes S, Armario A, Nadal R (2007). Litter size affects emotionality in adult male rats.. Physiol Behav.

[5] Faust IM, Johnson PR, Hirsch J (1980). Long-term effects of early nutritional experience on the development of obesity in the rat.. J Nutr.

[6] Aubert R, Suquet JP, Lemonnier D (1980). Long-term morphological and metabolic effects of early under- and over-nutrition in mice.. J Nutr.

[7] Ryan V, Wehmer F (1975). Effect of postnatal litter size on adult aggression in the laboratory mouse.. Dev Psychobiol.

[8] Schmidt I, Schoelch C, Ziska T, Schneider D, Simon E (2000). Interaction of genetic and environmental programming of the leptin system and of obesity disposition.. Physiol Genomics.

[9] Bouret SG, Simerly RB (2006). Developmental programming of hypothalamic feeding circuits.. Clin Genet.

[10] Bieswal F, Ahn MT, Reusens B, Holvoet P, Raes M (2006). The importance of catch-up growth after early malnutrition for the programming of obesity in male rat.. Obesity (Silver Spring).

[11] Fiorotto ML, Burrin DG, Perez M, Reeds PJ (1991). Intake and use of milk nutrients by rat pups suckled in small, medium, or large litters.. Am J Physiol.

[12] Cameron GN (1973). Effect of litter size on postnatal growth and survival in the desert woodrat.. J Mammal.

[13] Stockley P, Parker GA (2002). Life history consequences of mammal sibling rivalry.. Proc Natl Acad Sci U S A.

[14] Liu H, Zhong WQ, Wang DH (2003). Energy requirements during reproduction in female Brandt's voles (Microtus brandtii).. J Mammal.

[15] Zhang XY, Li YL, Wang DH (2008). Large litter size increases maternal energy intake but has no effect on UCP1 content and serum-leptin concentrations in lactating Brandt's voles (Lasiopodomys brandtii).. J Comp Physiol B.

[16] Friedman JM, Halaas JL (1998). Leptin and the regulation of body weight in mammals.. Nature.

[17] Zhang Y, Proenca R, Maffei M, Barone M, Leopold L (1994). Positional cloning of the mouse obese gene and its human homologue.. Nature.

[18] Bates SH, Stearns WH, Dundon TA, Schubert M, Tso AW (2003). STAT3 signalling is required for leptin regulation of energy balance but not reproduction.. Nature.

[19] Schwartz MW, Woods SC, Porte D, Seeley RJ, Baskin DG (2000). Central nervous system control of food intake.. Nature.

[20] Vaisse C, Halaas JL, Horvath CM, Darnell JE, Stoffel M (1996). Leptin activation of Stat3 in the hypothalamus of wild-type and ob/ob mice but not db/db mice.. Nat Genet.

[21] Bjorbaek C, Elmquist JK, Frantz JD, Shoelson SE, Flier JS (1998). Identification of SOCS-3 as a potential mediator of central leptin resistance.. Mol Cell.

[22] Wang Z, Zhou YT, Kakuma T, Lee Y, Kalra SP (2000). Leptin resistance of adipocytes in obesity: role of suppressors of cytokine signaling.. Biochem Biophys Res Commun.

[23] Bing C, Frankish HM, Pickavance L, Wang Q, Hopkins DF (1998). Hyperphagia in cold-exposed rats is accompanied by decreased plasma leptin but unchanged hypothalamic NPY.. Am J Physiol.

[24] Heldmaier G, Steinlechner S, Rafael J, Latteier B (1982). Photoperiod and ambient temperature as environmental cues for seasonal thermogenic adaptation in the Djungarian hamster, Phodopus sungorus.. Int J Biometeorol.

[25] Wang JM, Wang DH (2006). Comparison of nonshivering thermogenesis induced by dosages of norepinephrine from 3 allometric equations in Brandt s voles (*Lasiopodomys brandtii*).. Acta Theriologica Sinica.

[26] Zhao ZJ, Wang DH (2005). Short photoperiod enhances thermogenic capacity in Brandt's voles.. Physiol Behav.

[27] Zhang XY, Wang DH (2006). Energy metabolism, thermogenesis and body mass regulation in Brandt's voles (*Lasiopodomys brandtii*) during cold acclimation and rewarming.. Horm Behav.

[28] Lowry OH, Rosebrough NJ, Farr AL, Randall RJ (1951). Protein measurement with the Folin phenol reagent.. J Biol Chem.

[29] Li XS, Wang DH (2005). Regulation of body weight and thermogenesis in seasonally acclimatized Brandt's voles (*Microtus brandti*).. Horm Behav.

[30] Tang GB, Cui JG, Wang DH (2009). Role of hypoleptinemia during cold adaptation in Brandt's voles (*Lasiopodomys brandtii*).. Am J Physiol Regul Integr Comp Physiol.

[31] Koskela E (1998). Offspring growth, survival and reproductive success in the bank vole: a litter size manipulation experiment.. Oecologia.

[32] Velkoska E, Cole TJ, Dean RG, Burrell LM, Morris MJ (2008). Early undernutrition leads to long-lasting reductions in body weight and adiposity whereas increased intake increases cardiac fibrosis in male rats.. J Nutr.

[33] Rodel HG, Meyer S, Prager G, Stefanski V, Hudson R (2010). Litter size is negatively correlated with corticosterone levels in weanling and juvenile laboratory rats.. Physiol Behav.

[34] Remmers F, Schreuder MF, Gemke RJ, Delemarre-van de Waal HA (2008). Energy intake and resting energy expenditure in adult male rats after early postnatal food restriction.. Br J Nutr.

[35] Patterson CM, Bouret SG, Park S, Irani BG, Dunn-Meynell AA (2010). Large Litter Rearing Enhances Leptin Sensitivity and Protects Selectively Bred Diet-Induced Obese Rats from Becoming Obese.. Endocrinology.

[36] Howie GJ, Sloboda DM, Kamal T, Vickers MH (2009). Maternal nutritional history predicts obesity in adult offspring independent of postnatal diet.. J Physiol.

[37] Chen H, Simar D, Morris MJ (2009). Hypothalamic neuroendocrine circuitry is programmed by maternal obesity: interaction with postnatal nutritional environment.. PLoS One.

[38] Desai M, Gayle D, Babu J, Ross MG (2005). Programmed obesity in intrauterine growth-restricted newborns: modulation by newborn nutrition.. Am J Physiol Regul Integr Comp Physiol.

[39] Hales CN, Barker DJ (2001). The thrifty phenotype hypothesis.. Br Med Bull.

[40] Hales CN, Barker DJ (1992). Type 2 (non-insulin-dependent) diabetes mellitus: the thrifty phenotype hypothesis.. Diabetologia.

[41] Cannon B, Nedergaard J (2004). Brown adipose tissue: function and physiological significance.. Physiol Rev.

[42] Vaziri H, Dessain SK, Ng Eaton E, Imai SI, Frye RA (2001). hSIR2(SIRT1) functions as an NAD-dependent p53 deacetylase.. Cell.

[43] Smith TJ, Drummond GS, Kourides IA, Kappas A (1982). Thyroid hormone regulation of heme oxidation in the liver.. Proc Natl Acad Sci U S A.

[44] Klieverik LP, Coomans CP, Endert E, Sauerwein HP, Havekes LM (2009). Thyroid hormone effects on whole-body energy homeostasis and tissue-specific fatty acid uptake in vivo.. Endocrinology.

[45] Xiao XQ, Williams SM, Grayson BE, Glavas MM, Cowley MA (2007). Excess weight gain during the early postnatal period is associated with permanent reprogramming of brown adipose tissue adaptive thermogenesis.. Endocrinology.

[46] Rodrigues AL, de Moura EG, Passos MC, Trevenzoli IH, da Conceicao EP (2010). Postnatal early overfeeding induces hypothalamic higher SOCS3 expression and lower STAT3 activity in adult rats.. J Nutr Biochem.

[47] Vickers MH, Breier BH, Cutfield WS, Hofman PL, Gluckman PD (2000). Fetal origins of hyperphagia, obesity, and hypertension and postnatal amplification by hypercaloric nutrition.. Am J Physiol Endocrinol Metab.

[48] Hytinantti TK, Juntunen M, Koistinen HA, Koivisto VA, Karonen SL (2001). Postnatal changes in concentrations of free and bound leptin.. Arch Dis Child Fetal Neonatal Ed.

[49] Varma A, He J, Shin BC, Weissfeld LA, Devaskar SU (2004). Postnatal intracerebroventricular exposure to leptin causes an altered adult female phenotype.. Am J Physiol Endocrinol Metab.

[50] Kirk SL, Samuelsson AM, Argenton M, Dhonye H, Kalamatianos T (2009). Maternal obesity induced by diet in rats permanently influences central processes regulating food intake in offspring.. PLoS One.

[51] Passos MC, Toste FP, Dutra SC, Trotta PA, Lisboa PC (2009). Role of neonatal hyperleptinaemia on serum adiponectin and suppressor of cytokine signalling-3 expression in young rats.. Br J Nutr.

[52] Toste FP, de Moura EG, Lisboa PC, Fagundes AT, de Oliveira E (2006). Neonatal leptin treatment programmes leptin hypothalamic resistance and intermediary metabolic parameters in adult rats.. Br J Nutr.

[53] Trevenzoli IH, Rodrigues AL, Oliveira E, Thole AA, Carvalho L (2010). Leptin treatment during lactation programs leptin synthesis, intermediate metabolism, and liver microsteatosis in adult rats.. Horm Metab Res.

[54] Pereira-Toste F, Toste FP, Oliveira E, Trotta PA, Lisboa PC (2009). Early maternal hyperleptinemia programs adipogenic phenotype in rats.. Horm Metab Res.

[55] Stofkova A, Skurlova M, Kiss A, Zelezna B, Zorad S (2009). Activation of hypothalamic NPY, AgRP, MC4R, AND IL-6 mRNA levels in young Lewis rats with early-life diet-induced obesity.. Endocr Regul.

[56] Cerf ME, Williams K, van Rooyen J, Esterhuyse AJ, Muller CJ (2010). Gestational 30% and 40% fat diets increase brain GLUT2 and neuropeptide Y immunoreactivity in neonatal Wistar rats.. Int J Dev Neurosci.

[57] Chang GQ, Gaysinskaya V, Karatayev O, Leibowitz SF (2008). Maternal high-fat diet and fetal programming: increased proliferation of hypothalamic peptide-producing neurons that increase risk for overeating and obesity.. J Neurosci.

